# Assessment of Structural and Functional Abnormalities of the Myocardium and the Ascending Aorta in Fetus with Hypoplastic Left Heart Syndrome

**DOI:** 10.1155/2016/2616729

**Published:** 2016-02-15

**Authors:** Yan Jiang, Yali Xu, Jinliang Tang, Hongmei Xia

**Affiliations:** ^1^Department of Ultrasound, Xinqiao Hospital, Third Military Medical University, Chongqing 400037, China; ^2^Department of Pathology, Xinqiao Hospital, Third Military Medical University, Chongqing 400037, China

## Abstract

*Aims*. To detect anatomical and intrinsic histopathological features of the ascending aorta and left ventricular (LV) myocardium and evaluate right ventricular (RV) function in fetuses with hypoplastic left heart syndrome (HLHS).* Methods*. Twenty-five fetuses diagnosed with HLHS were followed up in the antenatal and postpartum periods. 12 necropsy heart specimens were analyzed for morphological and histological changes.* Results*. Prenatal echocardiography and pathologic anatomy displayed the typical characteristics of HLHS as a severe underdevelopment of the LV in the form of mitral stenosis or atresia or as aortic atresia or stenosis, with a decreased ratio of aortic diameter to pulmonary artery diameter (median of 0.49 with a range of 0.24 to 0.69, *p* ≤ 0.001) and a higher ratio of RV diameter to LV diameter (median of 2.44 with a range of 1.33 to 6.25, *p* ≤ 0.001). The RV volume, stroke volume, and cardiac output in HLHS fetuses were increased compared with the gestational age-matched normal controls (*p* < 0.01). Histological changes in the 12 HLHS specimens included LV myocardial fibrosis, aortic elastic fragmentation, and fibrosis.* Conclusions*. In addition to severe anatomical deformity, distinct histological abnormalities in the LV myocardium and aortic wall were identified in the fetuses with HLHS. RV function damage may be potentially exists.

## 1. Introduction

Hypoplastic left heart syndrome (HLHS) is a rare congenital heart disease that is characterized by the presentation of left ventricular (LV) inlet and outflow tract hypoplasia; it was first described by Lev in 1952 and termed “HLHS” by Noonan and Nadas [1, 2]. Recently, with the rapid development of ultrasound imaging and cardiovascular surgery techniques, strategies aimed at the clinical diagnosis, treatment, and prognostic prediction of HLHS have advanced substantially. Several studies have shown that a considerable proportion of patients with HLHS are now advancing to survival during the fetal period [3, 4]. Although a number of therapeutic options are currently available for families with a fetus that has been diagnosed with HLHS, the optimal treatment for this condition is a subject of debate. Moreover, the overall mortality rate remains high in these patients, and the quality of life for surviving patients with HLHS remains low. Despite some studies having reported relatively high 5-year survival rates in some patient series (50% to 69%, including those who underwent the Fontan procedure), the expected life expectancy for patients with HLHS remains short [5]. One hypothesis for the short lifespan of HLHS patients is that their progressive changes in cardiac function may be directly associated with progressive pathological changes in myocardial tissue. The use of ultrasound for evaluating myocardial characteristics in fetuses with HLHS has been reported; however, the histological features of fetuses with HLHS have rarely been described [6]. It would be of clinical significance to clarify the pathological features and intrinsic histological changes that are observed in fetuses with HLHS to gain a better understanding of the pathophysiological process that occurs during the fetal period in individuals with this disease. Additionally, postnatal outcome of fetuses with HLHS is mainly determined by right ventricular function. Therefore, the aim of the present study was to analyze the clinical, echocardiographic, anatomic, and histopathological features of fetuses with HLHS to determine whether intrinsic histological abnormalities are apparent during the fetal stages and to detect whether there are differences in the right ventricular function of fetuses with HLHS compared to healthy fetuses during gestation.

## 2. Methods

### 2.1. Patient Population

Twenty-five fetuses with LV hypoplasia, mitral stenosis or atresia, or aortic stenosis or atresia and 25 gestational age-matched normal controls were evaluated by prenatal echocardiography at Xinqiao Hospital, Chongqing, between February 1, 2006, and December, 31, 2012. We did not include fetuses with exclusive coarctation of the aorta (CoA). Through the kind efforts of the dedicated families who signed the donation agreements, 12 formalin-fixed heart specimens from HLHS fetuses (gestational age 23.9–34.3 weeks; 11 voluntary terminations of pregnancy and 1 fetal death in utero) and 6 normal control heart specimens (gestational age 22.6–32.1 weeks; inevitable abortion) were obtained from the Department of Pathology at Xinqiao Hospital. These specimens were suitable for both macroscopic and microscopic study. Sections of the LV myocardium and the aortic walls were labeled using hematoxylin and eosin (HE), Masson's trichrome, and elastic van Gieson (EVG) stains. Each section was examined by two independent pathologists. In instances where there was a discrepancy, the specimens were reevaluated by both pathologists and by a third pathologist to reach a consensus. Follow-ups in the antenatal and postpartum periods were performed for six live births; they included clinical interventions and changes in echocardiographic characteristics. Another seven cases were lost to follow-up. This study was performed in accordance with a protocol that was approved by the Xinqiao Hospital Committee for Clinical Investigations.

### 2.2. Fetal and Neonatal Echocardiographic Study

All fetal patients underwent at least 1 detailed prenatal echocardiogram, and 6 neonates received echo examinations each month following birth. Echocardiography was performed using a Philips IE33 unit (Philips Healthcare, Bothell, WA, USA), equipped with the latest version of QLAB analysis software, with S8-3, C5-1 probe, and X5-1 matrix probe. Multiple parameters, including cardiovascular anatomic structure and Doppler blood flow, were assessed. Specifically, the anatomic assessment included the measurement of the following structures, in accordance with the cardiac segmental approach: left atrial (LA) and right atrial (RA) dimensions, LV and right ventricular (RV) end-diastolic width diameters, and ascending aorta (AA) and pulmonary trunk (PT) diameters. The mitral valve (MV) was described as either anatomically normal or abnormal. Mitral valve stenosis (MS) was defined as a pathologically decreased diameter of the mitral valve annulus, where color Doppler imaging demonstrated minimal forward flow across the mitral valve. The diameter of the foramen ovale (FO) was also recorded. Cardiac Doppler evaluation included measurements of the MV and the tricuspid valve (TV) flow patterns, the MV and TV color Doppler regurgitant jet areas, and the velocities and flow patterns in the AA and PT. The direction of FO flow was further described. Real time three-dimensional echocardiography (RT-3DE) was used to measure RV end-diastolic volume (EDV), end-systolic volume (ESV), stroke volume (SV), cardiac output (CO), and ejection fraction (EF). All direct measurements were performed by a single experienced investigator. The investigator was blinded to the patient information when measuring these parameters.

### 2.3. Pathomorphological and Histological Analyses

When performing autopsies, the right atrial appendage and RV were opened along the lateral wall to the RV apex and along the interventricular septum to the pulmonary valve. The left side of the heart was similarly opened according to the path of blood flow [7]. Excised, full-thickness aortic walls and myocardial tissues from the LV anterior-lateral walls were obtained from 12 HLHS fetal heart specimens and were then studied. Aortal sections were examined under light microscopy to detect elastic fragmentation and fibrosis. Sections of the LV myocardium were also studied using light microscopy to detect myocardial fibrosis. Each of the investigators was blinded to patient information when analyzing the pathologic sections.

### 2.4. Data Analyses

The data are primarily presented in a descriptive fashion due to the small number of fetuses and infants included in our study. Statistical analyses were performed using a sample *t*-test to analyze ecological parameters between our echocardiographic results and normal reference standards. Data are presented as the median (range). Only *p* ≤ 0.01 was considered to be statistically significant.

## 3. Results

### 3.1. Patients

Twenty-five fetuses with HLHS, whose mothers underwent prenatal echocardiography at a gestational age of 28.0 ± 7.1 weeks (range 23.9 to 36.4 weeks) and 25 controls with a gestational age of 27.5 ± 2.9 weeks (range 24.3 to 35 weeks) were included in our study. None of the patients had a confirmed family history of HLHS. During follow-up, 11 parents elected to terminate the pregnancy after considering the poor prognosis for a fetal heart condition (9 males and 2 females). One fetus died at 32.6 weeks (male). Six of the fetuses were born alive and presented to Xinqiao Hospital due to progressive heart failure, delayed development, or pulmonary infection at a median age of 12.5 days (range 1.0 to 35.0 days). These six infants were all male. One died at 126 days of age, 1 day after a selective angiocardiography catheterization was performed during hospitalization. One died at 89 days of age, one week after computed-tomography angiography was performed. There were 4 twins (2 sets). Of these, 1 set of twins died (one twin at 18 days old and the other at 135 days old, one week after a pericardium patch was applied during an operation to widen the left ventricular outflow tract and CoA). The other set of twins was healthy (Table 1).

### 3.2. Echocardiography

**Figure 1:**
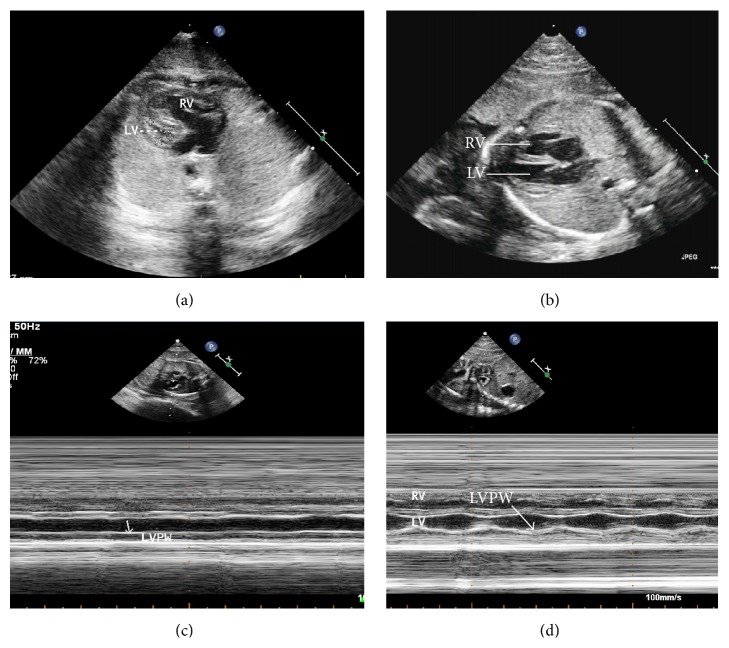
HLHS fetal echocardiogram images at 33 weeks of gestation. Fetal echocardiogram in a 4-chamber view, demonstrating that both the end-diastolic length and the diameter of left ventricle (LV) decreased (a) as compared with the normal heart in same gestational age (b), while the right ventricle (RV) diameter increased (a), resulting in a notably higher diameter ratio of the RV to LV. M mode echocardiogram image showed the poor contractility of LV (c, white arrow) as compared with the normal heart (d, white arrow). LV: left ventricle; RV: right ventricle; and LVPW: left ventricular posterior wall.

The results of two-dimensional and Doppler echocardiography performed on 18 fetuses with HLHS during the follow-up period are summarized in Table 2. The most notable abnormality was a severely underdeveloped LV, which was present in all of the patients in the form of mitral stenosis or atresia or as aortic atresia or stenosis. Color Doppler flow images displayed ductus arteriosus-dependent retrograde flow in the transverse aortic arch (Video 1 in Supplementary Material available online at http://dx.doi.org/10.1155/2016/2616729), no blood flow through the mitral valve during diastolic filling (Video 2), a restrictive atrial septum bulging from left to right, or an intact atrial septum. The ratio of the diameter of the aortic root to the diameter of the pulmonary artery was decreased, with a median of 0.49 and a range of 0.24 to 0.69, compared to the normal range at the same gestational age of 0.75 to 1.02 [8]. The LV diastolic diameter (LVDd) was significantly smaller than in normal hearts (Figures 1(a) and 1(b)), resulting in a markedly higher ratio of the diameter of the RV to the diameter of the LV, with a median of 2.44 and a range of 1.33 to 6.25. The normal range at the same gestational age is 0.93 to 1.03 [9]. The RV EDV, ESV, SV, and CO in HLHS fetuses are increased compared with the normal controls (*p* < 0.01). The RV EF showed no significant difference between HLHS fetuses and the controls (*p* > 0.05) (Table 3). In the 12 aborted fetuses included in the study, the LV showed poor contractility when compared to normal hearts (Figures 1(c) and 1(d)).

**Figure 2:**
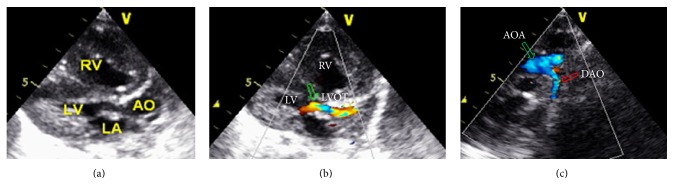
Infant echocardiogram images at 125 days. Echocardiogram in a left ventricular long axis view, demonstrating severe left ventricular outflow tract (LVOT) obstruction and biventricular hypertrophy (a). Doppler color flow image, displaying turbulence flow through LVOT (b, green arrow). Doppler color flow image in the suprasternal long axis view of the aortic arch, demonstrating low velocity laminar flow through the coarctation of the DAO (c, red arrow). AO: aorta; LA: left atrium; LV: left ventricle; RV: right ventricle; LVOT: left ventricular outflow tract; AOA: aortic arch; and DAO: descending aortic.

Six fetuses had either mild or severe left ventricular outflow tract (LVOT) obstruction. During the postnatal follow-up of the infants, one presented with progressive biventricular hypertrophy within a short period of time after birth (from 6 mm to 12 mm over three months) (Figures 2(a) and 2(b)); this patient also showed severe coarctation in the aortic sinus of the pipe junction, the ascending aorta, and the aortic isthmus. Color Doppler imaging demonstrated a decreased velocity across the CoA (from 3.8 m/sec to 1.92 m/sec) that was accompanied by an increased degree of biventricular hypertrophy over three months (Figure 2(c)). Furthermore, four fetuses had mild or moderate mitral regurgitation. Doppler recordings of peak systolic flow velocity were obtained at an insonation angle of less than 15 degrees to the flow and without any angle corrections.

### 3.3. Pathomorphological and Microscopic Histological Findings

**Figure 3:**
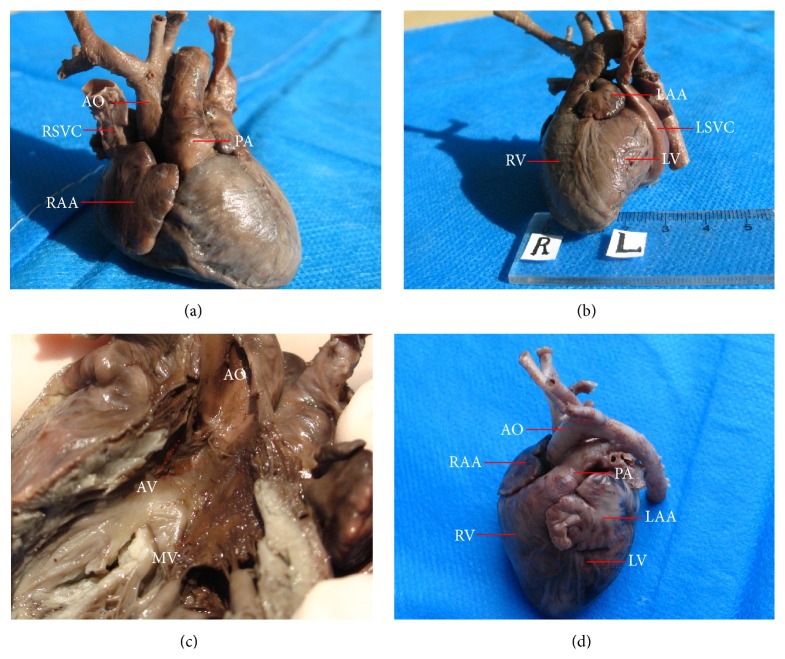
HLHS fetal autopsy heart specimen at 33.1 weeks of gestation. An autopsy heart specimen at 33.1 weeks gestation demonstrating decreased ratio of the diameter of the AO to that of the PA (a), severely underdeveloped LV and left atrial appendages in comparison with RV and right atrial appendages, with left superior vena cava (b), and aortic valve dysplasia, as well as an abnormal mitral valve along with shortened chordae and basal displacement of the papillary muscles, with the old clot deposition (c). A normal autopsy heart specimen at 33.5 weeks gestation as contrast (d). AO: aorta; RSVC: right superior vena cava; RAA: right atrial appendages; PA: pulmonary artery; LAA: left atrial appendages; RV: right ventricular; LSVC: left superior vena cava; LV: left ventricular; AV: aortic valve; and MV: mitral valve.

**Figure 4:**
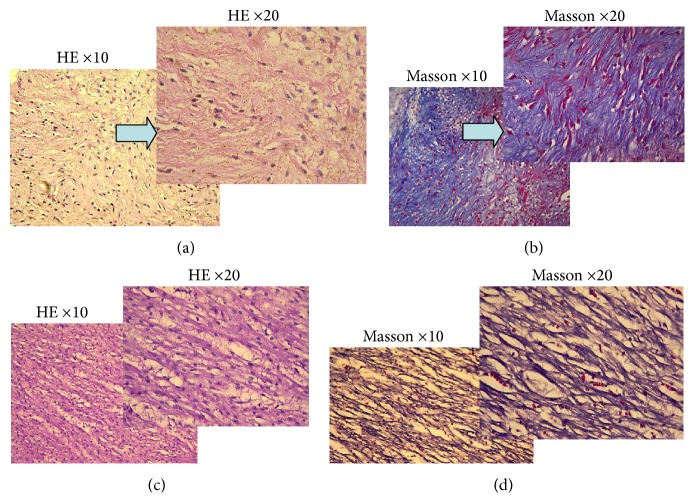
Histological abnormalities of left ventricular myocardium as compared with normal. Histological abnormalities from the LV myocardium of HLHS specimens presented with grade 3 fibrosis (a, b) compared to normal controls (c, d). Hematoxylin and eosin staining (a and c). Masson staining (b and d). Magnification ×10 (the bottom left of a, b, c, and d) and Magnification ×20 (the upper right of a, b, c, and d). HE: Hematoxylin and Eosin.

**Figure 5:**
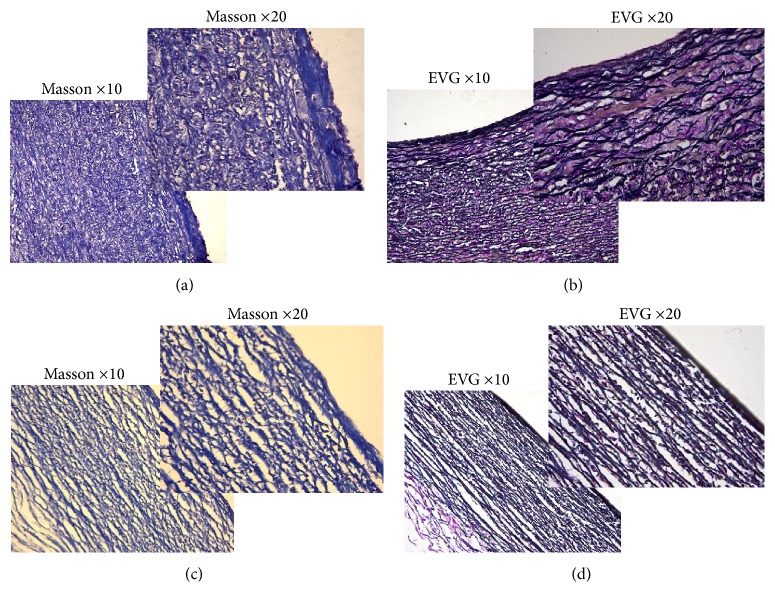
Aortic wall histopathology. Histological abnormalities from the aortic wall of HLHS specimens presented with fibrosis and elastic fragmentation (a and b) compared with normal controls (c and d). Masson staining (a and c). Elastic van Gieson staining (b and d). Magnification ×10 (the bottom left of a, b, c, and d) and magnification ×20 (the upper right of a, b, c, and d). EVG: elastic van Gieson.

Histomorphological and histopathological examinations of fetuses with HLHS were performed in the 12 induced cases. Among the autopsy specimens, 12 displayed a decreased ratio of the diameter of the aortic (AO) to the diameter of the pulmonary artery (PA) (Figure 3(a)), and all showed severely underdeveloped LV and left atrial appendages compared to the RV and right atrial appendages. They also displayed a left superior vena cava (Figure 3(b)), aortic valve dysplasia, and an abnormal mitral valve along with shortened chordae and basal displacement of the papillary muscles with old clot deposition (Figure 3(c)). Three specimens revealed the existence of aortic atresia and severe CoA in the transverse aortic arch. Seven cases exhibited a hypoplastic bicuspid aortic valve, three cases displayed a severe restrictive atrial septum, and another two cases showed a closed foramen ovale. Relative to the controls, histological abnormalities were detected in the LV myocardium and aortic wall specimens. The LV myocardium in the anterior wall of all of the patients with HLHS showed more extensive myofiber disarray and fibrosis (Figures 4(a) and 4(b)) than did the normal sections (Figures 4(c) and 4(d)). Histological abnormalities, including elastic fragmentation and fibrosis, were more common in the aortic walls of the HLHS heart specimens than among normal controls (Figure 5).

## 4. Discussion

HLHS has been recognized as a congenital heart disease with a high mortality rate following birth. However, little is known about the histopathological features of the aorta and LV myocardium during the fetal stages of the disease. This study reports the histopathological features of HLHS specimens from patients who presented with severe LV dysplasia, aortic stenosis or aortic atresia, mitral stenosis or mitral atresia, restrictive or intact atrial septum, and CoA. We hypothesized that these abnormalities might be a complex set of defects that should be considered as a distinct clinical entity.

### 4.1. Relationship of the Foramen Ovale to Left Heart Development in Fetuses

A small proportion of infants with HLHS have either an intact or a restrictive atrial septum. Their neonatal mortality is particularly high, even following successful atrial decompression [10]. An intact or restrictive atrial septum may result in pulmonary venous hypertension and abnormalities in pulmonary maturation that can persist even after the relief of atrial septal obstruction by septostomy [11]. In our study, there were five fetuses with an intact atrial septum and eight with a small foramen ovale (1–1.5 mm) that presented with a left-to-right instead of a right-to-left flow. These results indicate that the incidence of either an intact or restrictive atrial septum in fetuses is higher than the incidence in infants, which may be explained by the fact that these conditions cause a higher mortality rate during the neonatal period. Myocardial fibrosis and endocardial fibroelastosis were both present in patients during the fetal stage but were more substantial in fetuses with either a restrictive foramen ovale or an intact interatrial septum, which may either account for or coexist with a higher incidence of LV hypoplasia and premature AO growth. Theoretically, a prenatal intervention that includes opening the atrial septum and transcatheter fetal aortic valve dilation may be beneficial and may allow for the remodeling of the left heart architecture in utero during the later stages of pregnancy. This intervention has been reported as a viable therapeutic option in certain hospital centers [3].

However, the best timing for intervention and the possible feasibility of such a maneuver in a fetus remain speculative at this time. The favorable consequences, however, appear to increase the chances of successful postnatal biventricular physiology. Disappointingly, intervention during the second trimester has not been shown to improve the growth and development of LV dimensions [12]. The reasons for this are not clear, but it may be because the window for hyperplastic remodeling in humans is earlier than it is currently feasible to operate using conventional transcatheter techniques. Another possible reason may be attributed to the irreversible and progressive internal pathological changes in the myocardium, which might be related to genetic factors.

### 4.2. LV Hypoplasia and LV Hypertrophy

Normal cardiac morphogenesis requires blood-flow directed remodeling in addition to intrinsic patterning. Blood-flow directed remodeling contributes to the secondary development and differentiation of structures as a result of the effects of shear stress and the dynamics of blood flow within the structures [13]. LV hypoplasia is typically observed in association with LVOT obstruction. Secondary responses to LVOT obstruction are complex and may involve abnormal flow dynamics and shear stress that result in poor left ventricular development. Subsequent remodeling is likely affected by genetic factors, including alterations to intrinsic myocardial growth-signaling pathways. In addition, during the later stages of gestation, cardiomyocytes may lose the ability to undergo mitosis. Thus, ventricular hyperplasia can no longer occur, and remodeling is limited to muscular hypertrophy [14].

Cellular proliferation (hyperplasia) is confirmed to be an obligatory process during embryogenesis. In contrast, the developing human heart mainly results from cardiomyocyte hypertrophy and not from hyperplasia. Therefore, at some point during heart development, the ventricular muscle may undergo a switch in potential from hyperplasia to hypertrophy. This switch in myogenic potential may have important implications for ventricular remodeling in neonates with LVOT obstruction [14]. The results of our study indicated that the case (number 2) exhibited only mild ventricular hypertrophy during late gestation and after birth but showed severe LVOT obstruction. The degree of ventricular hypertrophy then doubled after three months (from 6 mm to 12 mm), and the patient displayed a rapid decline in cardiac function. Color Doppler imaging demonstrated a decreased velocity of the flow across the CoA that was accompanied by an increase in the degree of hypertrophy (Figure 2), which may reflect left heart failure exacerbation. In contrast to the typical hypertrophic response that accompanies a postnatal ventricular outflow obstruction, aortic banding in mid-gestation fetal sheep has been found to cause initial cardiomyocyte hypertrophy that is followed by a hyperplastic phase [15]. The same process occurred in another twin baby. In addition to LV outflow tract obstruction, there were severe sinus pipe connections and ascending aortic stenosis. We believe that there is no therapeutic effect from simple diameter widening of the CoA in patients with HLHS. In addition to changes in the vessel diameter, vascular wall elasticity and myocardial compliance and contractility also showed histopathological change in the myocardium and vascular wall. Left heart structures, including the mitral valve, aortic valve, and LV, are simply too small to support systemic circulation. In patients with left heart hypoplasia, altered myocardial compliance and contractility may result in the failure of biventricular repair.

### 4.3. LV Myocardial Histopathological Changes in Fetuses

In some congenital heart diseases, in addition to abnormal cardiac anatomy, myocardial histopathological changes can be discerned at an early developmental stage during the fetal period; such changes include noncompaction, fibrosis, and endocardial fibroelastosis (EFE) [16]. The changes in the histological characteristics of the myocardium and great vascular wall in fetuses with a single ventricle connected to the aortic coarctation are obvious and may be the critical cause that leads to progressive dysfunction in the postnatal heart [17]. Noncompaction and EFE have previously been detected by fetal echocardiography, and contemporary diagnostic criteria have been established [18–20]. As for HLHS, a rare type of complicated congenital heart disease, what happens to the LV myocardial histological characteristics during the fetal period? Postnatally, LV EFE has been demonstrated by MRI and histopathological examination in infants with HLHS [21]; however, myocardial histopathological changes in fetuses with HLHS have not been reported. This study tests the hypothesis that the myocardial defects are related to histological changes and confirms histological differences between normal and HLHS tissue.

### 4.4. RV Function

Because of severe left ventricular dysfunction being unable to maintain the circulation of blood perfusion in neonates with HLHS, RV is actually functional single ventricle and assumes the pulmonary circulation and blood circulation spring function. Therefore, an accurate assessment of right ventricular function in fetuses with HLHS would have given a more meaningful message on prenatal counseling, intrauterine intervention surgical treatment options, and prognosis after birth. Our results displayed that the RV volume, SV, and CO in HLHS fetuses were increased compared with the gestational age-matched normal controls, which indicates the compensated contraction of right ventricle. The RV EF showed no significant difference between HLHS fetuses and the controls. RV EF reflects the percentage of RV SV accounted for RV EDV. RV SV increases with the increase of RV EDV; both of them adapt to each other, thus leading to RV EF values remaining within a certain range of fluctuations in HLHS fetuses. Natarajan et al. showed that fetuses with endocardial fibroelastosis (EFE) of the left ventricle and HLHS presented with marked abnormal RV myocardial function by lower tricuspid *E*/*A* ratios, higher *E*/*e*′ ratios, and higher right ventricular myocardial performance indices [22]. Fetal HLHS is a rare heart disease with low incidence. Despite a long period of time, the number of cases with HLHS is still limited. Our small sample size may have reduced the effectiveness of the statistical analysis. The sensitivity and specificity remain to be confirmed in the future large multicenter study.

## 5. Limitations

Despite significant improvements in medical resources and therapies, the lifespan of patients with HLHS remains short. We hypothesized that the changes in cardiac function in these patients may be related to cardiac pathological changes. Cardiac morphogenesis and myocardial tissue structure changed significantly during the fetal period and in the first years after birth. Some related studies have reported the ultrasound and MRI characteristics of patients with HLHS, but few histopathological observations have been reported. With the support of the patients and Xinqiao Hospital, we were able to observe preliminary histopathological features in these patients. However, our case series contains only a limited number of patients. Large histopathological studies that include the right ventricle and pulmonary artery in HLHS specimens are needed to further understand the pathophysiologic features of HLHS patients.

## 6. Conclusion

In addition to severe anatomical deformity, distinct histological abnormalities in the LV myocardium and aortic wall were identified in the fetuses with HLHS. These changes suggest that structural abnormalities of the heart may be intrinsic in HLHS. RV function damage may potentially exist.

## Supplementary Material

Video 1: Doppler color flow image in the aortic arch view demonstrating a ductus arteriosus-dependent retrograde flow in the transverse aortic arch (white arrow).
Video 2: Doppler color flow image in a 4-chamber view demonstrating mitral atresia without blood filling, while blood flow filling normally through the tricuspid during diastole.

## Figures and Tables

**Table 1:** Clinical characteristics and follow-up data of the 6 fetuses who were born alive.

Fetus	GA at presentation (weeks)	GA at birth (weeks)	Gender	Postnatal intervention or traumatic examination	Follow-up
1 (twin 1)	32.4	38.6	Male	None	Death at 18 days
2 (twin 1)	32.4	38.6	Male	Pericardium patch widened operation in CoA	Death at 135 days (1 week after the operation)
3 (twin 2)	31.9	38.7	Male	None	Alive, 6 months
4	26.7	39.4	Male	Selective angiocardiography catheter	Death at 126 days (1 day after angiocardiography)
5 (twin 3)	34.6	38.2	Male	None	Alive, 5 months
6	28.9	39.1	Male	CT angiography	Death at 89 days (1 week after CT angiography)

GA: gestational age; CoA: coarctation of the aorta.

**Table 2:** Echocardiographic details of fetuses with HLHS in follow-up (*n* = 18).

FO (*n*)	
Normal	5
Restrictive	8
Intact	5
MV (*n*)	
Stenosis	11
Atresia	7
RV/LV diameter ratio	2.44 (1.33 to 6.25)^*∗*^
AV (*n*)	
Stenosis	14
Atresia	4
AO/PA diameter ratio	0.49 (0.24 to 0.69)^*∗*^

Values are presented as number (*n*) and median (range).

^*∗*^ *p*≦0.001 by 1-sample  *t*-test.

FO: foramen ovale; LV: left ventricular; RV: right ventricular; TV: tricuspid valve.

**Table 3:** Comparison of RV volume and RV function parameters between HLHS (*n* = 25) and the gestational age-matched normal controls (*n* = 25).

Groups	GA (weeks)	RV EDV (mL)	RV ESV (mL)	RV SV (mL)	RV CO (mL/min)	RV EF (%)
HLHS (*n* = 25)	28.0 ± 7.1	3.92 ± 1.29^*∗*^	1.55 ± 0.68^*∗*^	2.42 ± 0.56^*∗*^	352.41 ± 116.86^*∗*^	60.93 ± 4.58
Controls (*n* = 25)	27.5 ± 2.9	3.36 ± 1.51	1.23 ± 0.51	2.11 ± 0.87	325.35 ± 130.15	61.65 ± 4.36

^*∗*^ *p* < 0.01, versus normal control group.

GA: gestational age; RV: right ventricular; HLHS: hypoplastic left heart syndrome; EDV: end-diastolic volume; ESV: end-systolic volume; SV: stroke volume; CO: cardiac output; EF: ejection fraction.
